# ELP3 stabilizes c-Myc to promote tumorigenesis

**DOI:** 10.1093/jmcb/mjad059

**Published:** 2023-09-28

**Authors:** Wentao Zhao, Cong Ouyang, Chen Huang, Jiaojiao Zhang, Qiao Xiao, Fengqiong Zhang, Huihui Wang, Furong Lin, Jinyang Wang, Zhanxiang Wang, Bin Jiang, Qinxi Li

**Affiliations:** State Key Laboratory of Cellular Stress Biology, School of Life Sciences, Faculty of Medicine and Life Sciences, Xiamen University, Xiamen 361102, China; Department of Neurosurgery and Department of Neuroscience, Fujian Key Laboratory of Brain Tumors Diagnosis and Precision Treatment, Xiamen Key Laboratory of Brain Center, the First Affiliated Hospital of Xiamen University, School of Medicine, Xiamen University, Xiamen 361003, China; State Key Laboratory of Cellular Stress Biology, School of Life Sciences, Faculty of Medicine and Life Sciences, Xiamen University, Xiamen 361102, China; State Key Laboratory of Cellular Stress Biology, School of Life Sciences, Faculty of Medicine and Life Sciences, Xiamen University, Xiamen 361102, China; State Key Laboratory of Cellular Stress Biology, School of Life Sciences, Faculty of Medicine and Life Sciences, Xiamen University, Xiamen 361102, China; State Key Laboratory of Cellular Stress Biology, School of Life Sciences, Faculty of Medicine and Life Sciences, Xiamen University, Xiamen 361102, China; State Key Laboratory of Cellular Stress Biology, School of Life Sciences, Faculty of Medicine and Life Sciences, Xiamen University, Xiamen 361102, China; State Key Laboratory of Cellular Stress Biology, School of Life Sciences, Faculty of Medicine and Life Sciences, Xiamen University, Xiamen 361102, China; State Key Laboratory of Cellular Stress Biology, School of Life Sciences, Faculty of Medicine and Life Sciences, Xiamen University, Xiamen 361102, China; State Key Laboratory of Cellular Stress Biology, School of Life Sciences, Faculty of Medicine and Life Sciences, Xiamen University, Xiamen 361102, China; Department of Neurosurgery and Department of Neuroscience, Fujian Key Laboratory of Brain Tumors Diagnosis and Precision Treatment, Xiamen Key Laboratory of Brain Center, the First Affiliated Hospital of Xiamen University, School of Medicine, Xiamen University, Xiamen 361003, China; State Key Laboratory of Cellular Stress Biology, School of Life Sciences, Faculty of Medicine and Life Sciences, Xiamen University, Xiamen 361102, China; State Key Laboratory of Cellular Stress Biology, School of Life Sciences, Faculty of Medicine and Life Sciences, Xiamen University, Xiamen 361102, China

**Keywords:** ELP3, c-Myc, FBXW7, Elongator

## Abstract

ELP3, the catalytic subunit of the Elongator complex, is an acetyltransferase and associated with tumor progression. However, the detail of ELP3 oncogenic function remains largely unclear. Here, we found that ELP3 stabilizes c-Myc to promote tumorigenesis in an acetyltransferase-independent manner. Mechanistically, ELP3 competes with the E3-ligase FBXW7β for c-Myc binding, resulting in the inhibition of FBXW7β-mediated ubiquitination and proteasomal degradation of c-Myc. ELP3 knockdown diminishes glycolysis and glutaminolysis and dramatically retards cell proliferation and xenograft growth by downregulating c-Myc, and such effects are rescued by the reconstitution of c-Myc expression. Moreover, ELP3 and c-Myc were found overexpressed with a positive correlation in colorectal cancer and hepatocellular carcinoma. Taken together, we elucidate a new function of ELP3 in promoting tumorigenesis by stabilizing c-Myc, suggesting that inhibition of ELP3 is a potential strategy for treating c-Myc-driven carcinomas.

## Introduction

Elongator, the multi-subunit complex, was first purified from ribonucleic acid (RNA) polymerase II holoenzyme in 1999 (Otero et al., 1999). The original study provided evidence that Elongator is engaged in transcriptional elongation through promoting histone acetylation in the nuclear (Hawkes et al., 2002; Winkler et al., 2002). However, subsequent studies show that most of Elongator is located in the cytoplasm and plays an important role in transfer RNA (tRNA) modification by adding the 5-methoxycarbonylmethyl (mcm^5^) and 5-carbamoylmethy (ncm^5^) group to the wobble base of tRNAs (Dauden et al., 2019; Lin et al., 2019). The Elongator complex consists of six subunits (ELP1–ELP6) and ELP3 is the catalytic subunit harboring a lysine acetyltransferase (KAT) domain and a radical S-adenosyl-L-methionine (rSAM) domain (Fellows et al., 2000; Krogan and Greenblatt, 2001; Winkler et al., 2001).

Accumulating lines of evidence show that ELP3 is closely associated with tumor progression. It is reported that ELP3 plays a key role in hypoxia-inducible factor-1α (HIF-1α) translation by a codon-specific mechanism and promotes resistance to serine/threonine-protein kinase B-Raf (BRAF) inhibition in melanoma (Rapino et al., 2018). Knockdown of ELP3 inhibits the oncoprotein DEK translation, which reduced LEF1 IRES-dependent translation and tumor invasion (Delaunay et al., 2016). ELP3 is also found to be required for Sox9 translation and maintenance of a subset of Lgr5^+^/Dclk1^+^/Sox9^+^ cells which is crucial for Wnt-driven intestinal tumor initiation and radiation-induced regeneration (Ladang et al., 2015). In hematopoietic system, loss of ELP3 activates a p53-dependent antitumor checkpoint (Rosu et al., 2021). Interestingly, protein substrates were also reported. It has been shown that ELP3 acetylates glucose-6-phosphate dehydrogenase (G6PD) to modulate nicotinamide adenine dinucleotide phosphate (NADPH) homeostasis during oxidative stress (Wang et al., 2014). In addition, ELP3 acetylates α-tubulin to support radial migration and branching of cortical projection neurons (Creppe et al., 2009). In conclusion, carcinogenic function of ELP3 described mainly relies on its acetyltransferase activity. However, little is known about the function of ELP3 other than acetyltransferase in tumor progression.

c-Myc is a transcriptional factor with the basic helix–loop–helix (HLH) leucine zipper (b-HLH-LZ) domain (Hayward et al., 1981; Vennstrom et al., 1982). c-Myc is extensively overexpressed in a wide range of human tumors and plays a key role in malignant transformation (Dang, 2012). The transcriptional factor of c-Myc functions as heterodimer with Max and binds to E-box element (CACGTG) of downstream genes to involve in various cellular processes, including cell proliferation, metabolism, protein synthesis, and cell cycle progression (Amati et al., 1993). c-Myc is strictly regulated at multiple levels. It has been reported that Wnt, Hedgehog, Notch, and JAK/STAT signaling pathways promote c-Myc transcription (He et al., 1998; Kiuchi et al., 1999; Palomero et al., 2006; Sharma et al., 2006; Sansom et al., 2007), and c-Myc translation is strictly controlled by mTORC1/S6K1 and MAPK/HNRPK pathways (Notari et al., 2006; Wall et al., 2008; Csibi et al., 2014). In particular, the half-life of c-Myc is extremely short (∼30 min), highlighting the importance of protein stability in the c-Myc regulation. FBXW7 acts as the major E3-ligase and binds to MBI motif, which facilitates c-Myc ubiquitination and proteasomal degradation (Yada et al., 2004; Zhang et al., 2019). Consistent with the oncogenic function of c-Myc, the genomic region that harbors FBXW7 gene is found to be deleted in >30% of human cancers (Spruck et al., 2002).

In this study, we investigated the role of ELP3, involving the regulation of c-Myc stability, in tumor progression.

## Results

### ELP3 is overexpressed and correlated with tumorigenesis

**Figure 1:**
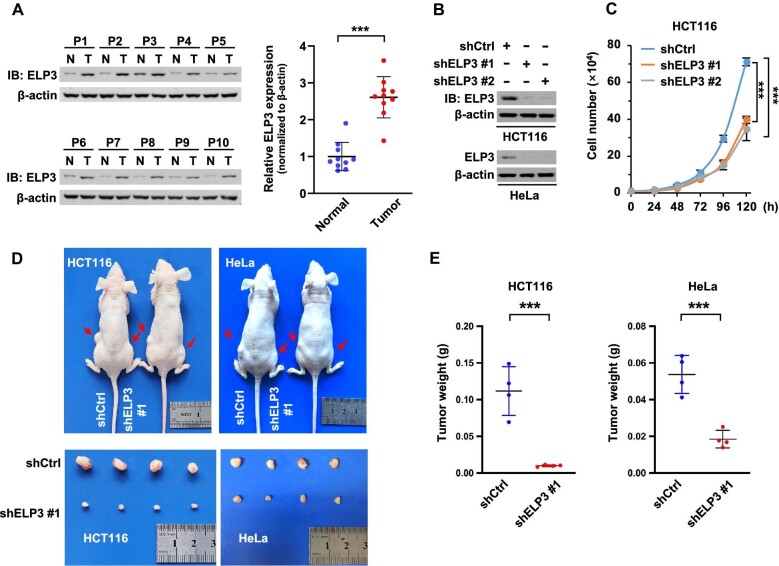
ELP3 is overexpressed and correlated with tumorigenesis. (**A**) Clinical tissue samples were collected from colorectal cancer patients (P, patient, *n* = 10; N, normal tissue; T, tumor tissue). ELP3 expression was determined by western blotting (β-actin as control) and quantitatively analyzed according to the optical density integrity (ODI). (**B**) ELP3-knockdown HCT116 and HeLa cells (by two independent shRNAs, shELP3 #1 and shELP3 #2; shCtrl, pLKO.1-based scramble shRNA as control) were examined by western blotting 3 days after infection. (**C**) HCT116 cells (1 × 10^4^) were seeded in 6-well plates, and cell numbers were counted at the indicated time points (*n* = 3). (**D** and **E**) ELP3-knockdown or control HCT116 and HeLa cells were subcutaneously injected into nude mice (2 × 10^6^ cells) for xenograft tumor formation assay (*n* = 4). Two representative mice per group are shown in **E**. Data are shown as mean ± SD. Unpaired Student's *t*-test. ***P < 0.001.

Previous studies provided evidence that ELP3 is involved in tumor progression. In line with these studies, ELP3 expression is significantly increased in clinical colorectal cancer tissues compared with normal tissues (Figure 1A). To evaluate the importance of ELP3 in cell proliferation, we knocked down ELP3 by two independent short hairpin RNA (shRNA) in HCT116 and HeLa cells (Figure 1B), and determined cell growth through morphology (Supplementary Figure S1A), cell counting (Figures 1C; Supplementary Figure S1B), and cell viability via CCK-8 assays (Supplementary Figure S1C and D). Colony formation numbers were reduced in ELP3-knockdown cells (Supplementary Figure S1E and F). We constructed ELP3-knockdown HCT116 and HeLa cells followed by subcutaneous transplantation. Xenograft assay showed that ELP3 knockdown extensively diminished tumor size (Figure 1D and E). In conclusion, ELP3 is overexpressed and correlated with tumorigenesis.

### ELP3 interacts with c-Myc

**Figure 2:**
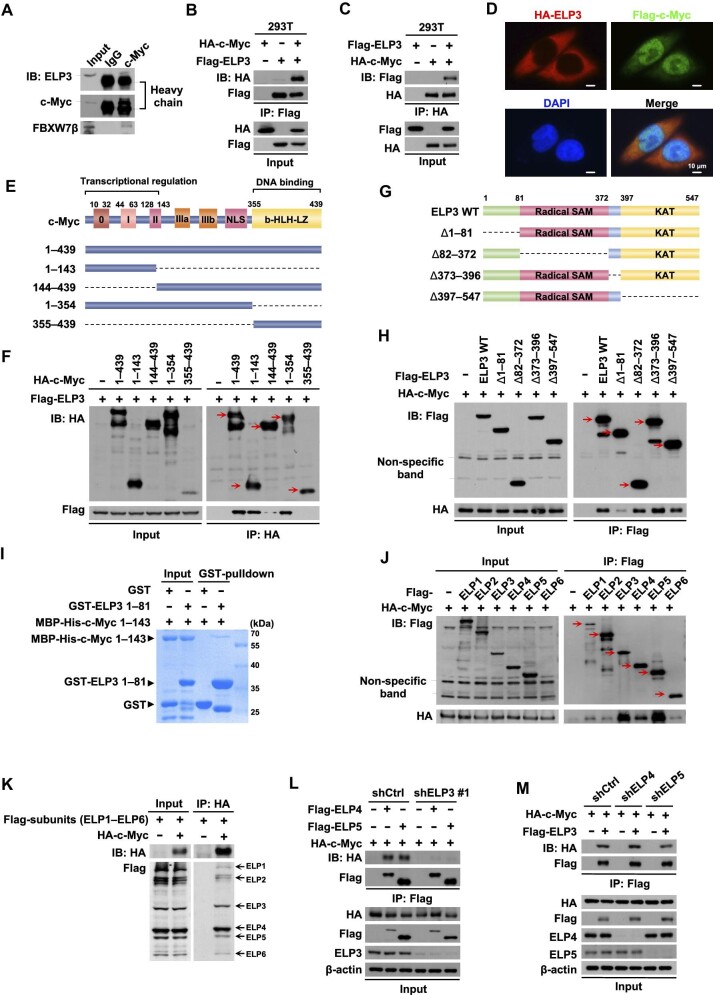
ELP3 interacts with c-Myc. (**A**) Endogenous IP assay was performed with rabbit anti-c-Myc antibody (rabbit IgG as control), followed by immunoblotting (IB) for ELP3, c-Myc, and FBXW7β. (**B** and **C**) HA-c-Myc and Flag-ELP3 were co-transfected into HEK-293T cells. Reciprocal co-IP assays were performed with anti-Flag M2 beads (**B**) and anti-HA A/G beads (**C**). Total cell lysates (Input) and the immunoprecipitates (IP) were analyzed by IB for HA and Flag. (**D**) Subcellular locations of ELP3 and c-Myc were analyzed by immunofluroscence staining in HeLa cells expressing Flag-c-Myc (green, 488) and HA-ELP3 (red, 555). Scale bar, 10 μm. (**E**) A schematic diagram of c-Myc. 0, box-0; I, box-I; II, box-II; IIIa, box-IIIa; IIIb, box-IIIb; NLS, nuclear localization sequence. (**F**) HA-tagged c-Myc with distinct domain deletion was constructed and co-transfected with Flag-ELP3 into HEK-293T cells, followed by IP for Flag and IB for HA to map the interacting-domain of c-Myc with ELP3. (**G**) A schematic diagram of ELP3. (**H**) Flag-tagged ELP3 with distinct domain deletion was constructed and co-transfected with HA-c-Myc into HEK-293T cells, followed by IP for HA and IB for Flag to map the interacting-domain of ELP3 with c-Myc. (**I**) GST, GST-ELP3 1–81, and MBP-HIS-c-Myc 1–143 were purified from BL21 *E. coli* competent cells. GST-pulldown assay was performed, followed by SDS–PAGE and Coomassie brilliant blue staining. (**J**) Six Elongator subunits (ELP1–ELP6) were individually cloned and co-transfected with HA-c-Myc into HEK-293T cells, followed by IP for Flag and IB for HA to determine the interaction between c-Myc and each Elongator subunit. (**K**) The six subunits (ELP1–ELP6) of the Elongator complex were co-transfected with HA-c-Myc into HEK-293 cells, followed by IP for HA and IB for Flag. (**L**) Flag-ELP4 or Flag-ELP5 was co-transfected with HA-c-Myc into control or ELP3-knockdown HEK-293T cells, followed by IP for Flag and IB for HA to analyze the interaction between ELP4 (or ELP5) and c-Myc. (**M**) HA-c-Myc and Flag-ELP3 were co-transfected into control, ELP4-knockdown, or ELP5-knockdown HEK-293T cells, followed by IP for Flag and IB for HA and Flag.

ELP3 was detected in the immunoprecipitate of c-Myc by endogenous immunoprecipitation (IP) assay (Figure 2A). We further confirmed the interaction between ELP3 and c-Myc by conducting reciprocal co-IP assays with overexpressed protein (Figure 2B and C). Additionally, a clear co-localization between ELP3 and c-Myc was observed in the cytosol, despite the fact that most of the c-Myc protein was localized in nuclear (Figure 2D). To determine the binding region of c-Myc to ELP3, various deletion mutants of c-Myc were constructed. The transcriptional regulation region of c-Myc, namely 1–143 amino acids (abbreviated as 1–143 a.a.), was required for its interaction with ELP3 (Figure 2E and F). Reciprocally, depletion of 1–81 amino acids of ELP3 disrupted the interaction with c-Myc (Figure 2G and H). In addition, ectopically expressed c-Myc 1–143 truncation could be co-precipitated by ELP3 1–81 truncation in an *in vitro* GST-pulldown assay, demonstrating a direct interaction of ELP3 and c-Myc (Figure 2I). However, it is not clear whether the interaction of ELP3 with c-Myc depends on entire Elongator complex. The six subunits of Elongator complex as described were cloned and co-expressed with c-Myc respectively, followed by IP assay. As shown in Figure 2J, HA-tagged c-Myc was detected in the immunoprecipitate of all six subunits. Subsequently, the six Flag-tagged subunits, ELP1–ELP6, were transfected all together with HA-c-Myc and all subunits were enriched in the immunoprecipitate of HA-c-Myc (Figure 2K). Clearly, ELP3, ELP4, and ELP5 had stronger interactions with c-Myc (Figure 2J). Knockdown of ELP3 completely abolished the interaction between ELP4 and ELP5 with c-Myc (Figure 2L), indicating that the interaction of ELP4 and ELP5 with c-Myc depends on the existence of ELP3. Consistent with the result of GST-pulldown assay, the interaction between ELP3 and c-Myc occurs in ELP4/5-independent mechanism (Figure 2M). Taken together, our results demonstrate that ELP3 interacts with c-Myc.

### ELP3 stabilizes c-Myc

**Figure 3:**
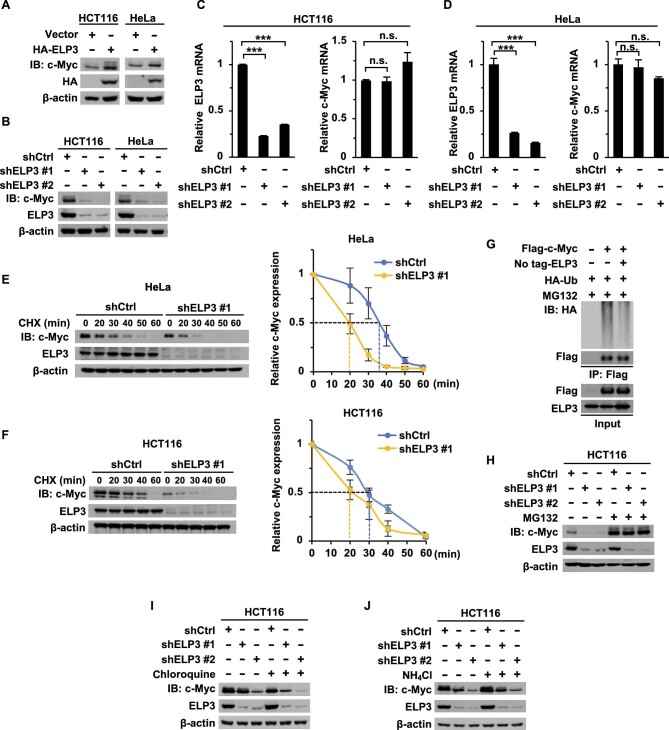
ELP3 stabilizes c-Myc. (**A**) Endogenous c-Myc protein expression in control or HA-ELP3-overexpressing HCT116 (left) and HeLa (right) cells was determined by western blotting. (**B**–**D**) ELP3 and c-Myc protein expression was determined by western blotting (**B**) and ELP3 and c-Myc mRNA levels were determined by qRT-PCR (**C** and **D**) in control or ELP3-knockdown HCT116 and HeLa cells. (**E** and **F**) Control or ELP3-knockdown HeLa and HCT116 cells were treated with CHX (50 μg/ml) for the indicated time. Protein expression was determined by western blotting and quantitatively analyzed with three biological replicates for c-Myc half-life. (**G**) Flag-c-Myc, no tag-ELP3, and HA-Ub were transfected as indicated into HEK-293T cells. After 16 h, the cells were treated with MG132 (20 μM) for 8 h, followed by IP for Flag and IB for HA to detect the ubiquitination of Flag-c-Myc. (**H**–**J**) HCT116 cells were treated with the proteasomal inhibitor MG132 (20 μM, **H**), chloroquine (50 μg/ml, **I**), or NH_4_Cl (25 mM, **J**) for 8 h. ELP3 and c-Myc protein expression was detected by western blotting. Data are shown as mean ± SD of at least three independent experiments. Unpaired Student's *t*-test. n.s., no significance (*P* ≥ 0.05); ***P < 0.001.

After clarification of the interaction, we continued to investigate the regulatory effect of ELP3 on c-Myc expression. Overexpression of ELP3 in HCT116 and HeLa cells increases c-Myc expression (Figure 3A). On the contrary, knockdown of ELP3 decreased c-Myc protein (Figure 3B) but not c-Myc messenger RNA (mRNA) (Figure 3C and D). To further explore whether ELP3 affects the c-Myc protein stability, we treated cells with cyclohexane (CHX) to inhibit protein synthesis and found that the half-life of c-Myc protein is significantly shortened to 20 min in ELP3-knockdown cells (Figure 3E and F). Consistently, the ubiquitination of c-Myc is dramatically decreased when cells were transfected with ELP3 (Figure 3G). Furthermore, the treatment with the proteasomal inhibitor MG132 abolished the effect of ELP3 knockdown on c-Myc expression (Figure 3H) but not the treatments with lysosome inhibitors (chloroquine and NH_4_Cl) (Figure 3I and J). Conclusively, ELP3 interacts with c-Myc to stabilize its expression.

### ELP3 promotes c-Myc expression via an acetyltransferase-independent mechanism

**Figure 4:**
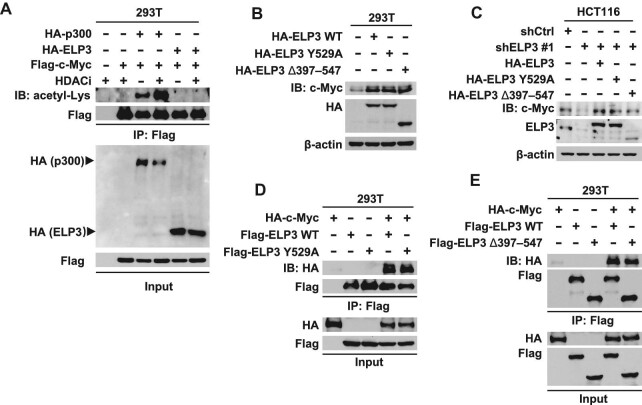
ELP3 promotes c-Myc expression via an acetyltransferase-independent mechanism. (**A**) Flag-c-Myc was co-transfected with HA-p300 or HA-ELP3 into HEK-293T cells. After 24 h, the cells were treated with HDACi (NAM: 30 mM; vorinostat: 3 μM; panolinostat: 5 μM) for 6 h, followed by IP for Flag and IB for acetyl-Lys to detect the acetylation of c-Myc. (**B**) HA-ELP3 WT, HA-ELP3 Y529A (mutant deficiency in acetyltransferase activity), and HA-ELP3 Δ397–547 (acetyltransferase domain deletion form) were transfected into HEK-293T cells. Endogenous c-Myc expression was determined by western blotting. (**C**) HA-tagged ELP3 WT, ELP3 Y529A, or ELP3 Δ397–547 was reconstituted in ELP3-knockdown HCT116 cells, and c-Myc protein expression was determined by western blotting. (**D** and **E**) HA-c-Myc was co-transfected with Flag-ELP3 WT, Flag-ELP3 Y529A (**D**), or Flag-ELP3 Δ397–547 (**E**) into HEK-293T cells, followed by IP for Flag and IB for HA to determine the interaction between c-Myc and ELP3.

Previous studies reported that ELP3 functions as an acetyltransferase involved in protein acetylation and tRNA modification. And it is widely accepted that p300 is an acetyltransferase of c-Myc (Yada et al., 2004; Faiola et al., 2005). To test whether the acetyltransferase activity of ELP3 is indispensable for c-Myc stabilization, ELP3 and p300 were co-transfected with c-Myc. As shown in Figure 4A, c-Myc was significantly acetylated after transfection with p300 but not ELP3. ELP3-Y529A (a mutant deficient in acetyltransferase activity) and ELP3 Δ397–547 (a mutant with acetyltransferase domain deletion) were constructed. As shown in Figure 4B, both mutants lack of acetyltransferase activity could increase c-Myc expression. Then, wild-type ELP3 (ELP3 WT), ELP3 Y529A, and ELP3 Δ397–547 were reconstituted in ELP3-knockdown cells, showing that both mutans restored c-Myc expression as well as ELP3 WT (Figure 4C). Indeed, ELP3 interacts with c-Myc in an acetyltransferase-independent manner (Figure 4D and E). These data indicated that ELP3 promotes c-Myc expression via an acetyltransferase-independent mechanism.

### ELP3 binds to c-Myc and inhibits FBXW7β-mediated proteasomal degradation

**Figure 5:**
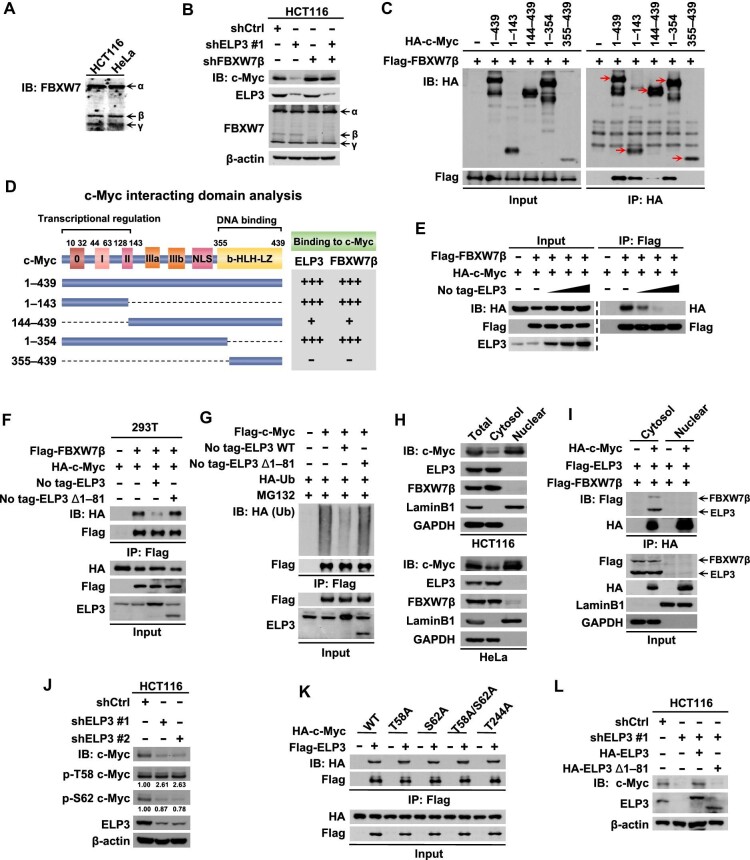
ELP3 binds to c-Myc and inhibits FBXW7β-mediated proteasomal degradation. (**A**) Three isoforms of FBXW7 in HCT116 and HeLa cells were determined by western blotting. (**B**) ELP3, c-Myc, and FBXW7 protein expression in control, ELP3-knockdown, FBXW7β-knockdown, or double-knockdown HCT116 cells was determined by western blotting. (**C**) HA-tagged c-Myc with distinct domain deletion was co-transfected with Flag-FBXW7β into HEK-293T cells, followed by IP for HA and IB for Flag to map the FBXW7β-interacting domain of c-Myc. (**D**) Analysis of c-Myc domains interacting with ELP3 and FBXW7. (**E**) Flag-FBXW7β (1 μg), HA-c-Myc (1 μg), and no tag-ELP3 (0.2, 0.5, and 1 μg) were transfected as indicated into HEK-293T cells, followed by IP for Flag and IB for HA to determine the interaction between FBXW7β and c-Myc. (**F**) Flag-FBXW7β and HA-c-Myc were co-transfected with no tag-ELP3 WT or no tag-ELP3 Δ1–81 as indicated into HEK-293 cells, followed by IP for Flag and IB for HA to determine the interaction between c-Myc and FBXW7β. (**G**) Flag-c-Myc, no tag-ELP3 WT or no tag-ELP3 Δ1–81, and HA-Ub were transfected as indicated into HEK-293T cells. After 16 h, the cells were treated with MG132 for 8 h, followed by IP for Flag and IB for HA to detect the ubiquitin of c-Myc. (**H**) ELP3, c-Myc, and FBXW7β protein expression in total lysates, cytoplasmic extraction, or nuclear extraction of HCT116 and HeLa cells was assayed by western blotting to determine their subcellular localization. (**I**) HA-c-Myc, Flag-ELP3, and Flag-FBXW7β were transfected as indicated into HEK-293T cells. After 24 h, cell fractionation was performed, followed by IP for HA and IB for Flag to determine the interaction between c-Myc and ELP3 (FBXW7β) in the cytoplasm and nucleoplasm. (**J**) The phosphorylation of c-Myc (p-T58 and p-S62) in control or ELP3-knockdown HCT116 cells was determined by western blotting and quantitatively analyzed (ratio of p-T58/c-Myc or p-S62/c-Myc). (**K**) HA-tagged WT or mutant (T58, S62A, T58A/S62A, or T244A) c-Myc was constructed and co-transfected with Flag-ELP3 as indicated into HEK-293T cells, followed by IP for Flag and IB for HA. (**L**) HA-ELP3 WT or HA-ELP3 Δ1–81 was reconstituted in ELP3-knockdown HCT116 cells, and c-Myc protein expression was determined by western blotting.

The half-life of c-Myc is astonishingly short (∼30 min), indicating the central role of protein stability in the regulation of c-Myc. It has been reported that FBXW7, the major c-Myc ubiquitin ligase, has three isoforms (FBXW7α, FBXW7β, and FBXW7γ) (Yada et al., 2004; Zhang et al., 2019; Yumimoto and Nakayama, 2020), and FBXW7α is predominately localized in nucleoplasm and FBXW7γ is nucleolar, whereas FBXW7β is found in a cytoplasmic distribution (Welcker et al., 2004). Given the localization of ELP3 in cytoplasm, three FBXW7 isoforms were detected and FBXW7β (cytoplasmic isoform) is further knocked down, showing that ELP3 knockdown failed to decrease c-Myc expression in FBXW7β-knockdown cells (Figure 5A and B). Next, we investigated the c-Myc regions responsible for its binding to FBXW7β. The transcriptional regulation region (1–143 a.a.) of c-Myc was also crucial for interaction with FBXW7β (Figure 5C), suggesting that FBXW7β shares the same c-Myc interacting regions with ELP3 (Figure 5D). ELP3 competes with FBXW7β for binding to c-Myc (Figure 5E), but this competition between ELP3 and FBXW7β for c-Myc binding is diminished when 1–81 a.a. of ELP3 was deleted (Figure 5F). Consistently, expression of ELP3 extensively blocked the ubiquitination of c-Myc, while the ELP3 Δ1–81 mutant did not show any effect on c-Myc ubiquitination (Figure 5G). Subcellular fraction assays showed that ELP3 co-localized with FBXW7β in cytosol (Figure 5H), where c-Myc interacts with ELP3 or FBXW7β (Figure 5I). To further explore the detail of the competition of ELP3 with FBXW7β for c-Myc binding, we analyzed the phosphorylation status of c-Myc (p-T58 and p-S62) and found a significant decrease in phosphorylation of S62 (p-S62/c-Myc) and an increase in phosphorylation of T58 (p-T58/c-Myc) in ELP3-knockdown cells (Figure 5J). These results suggest that the binding of ELP3 to c-Myc may abolish the phosphorylation of c-Myc at T58 and further disrupt the recognition of FBXW7β. Intriguingly, the interaction between ELP3 and c-Myc was not affected by the phosphorylation of c-Myc, which is required for c-Myc ubiquitination and acts to engage FBXW7 (Yada et al., 2004; Welcker et al., 2022; Figure 5K). ELP3 Δ1–81, a mutant incapable of interacting with c-Myc, fails to stabilize c-Myc (Figure 5L). In summary, these data clearly demonstrated that ELP3 binds to c-Myc via its 1–81 a.a. region to inhibit the phosphorylation of c-Myc at T58 and consequently blocks its proteasomal degradation mediated by FBXW7β.

### ELP3 stabilizes c-Myc to facilitate metabolic reprogramming, cell proliferation, and tumorigenesis

**Figure 6:**
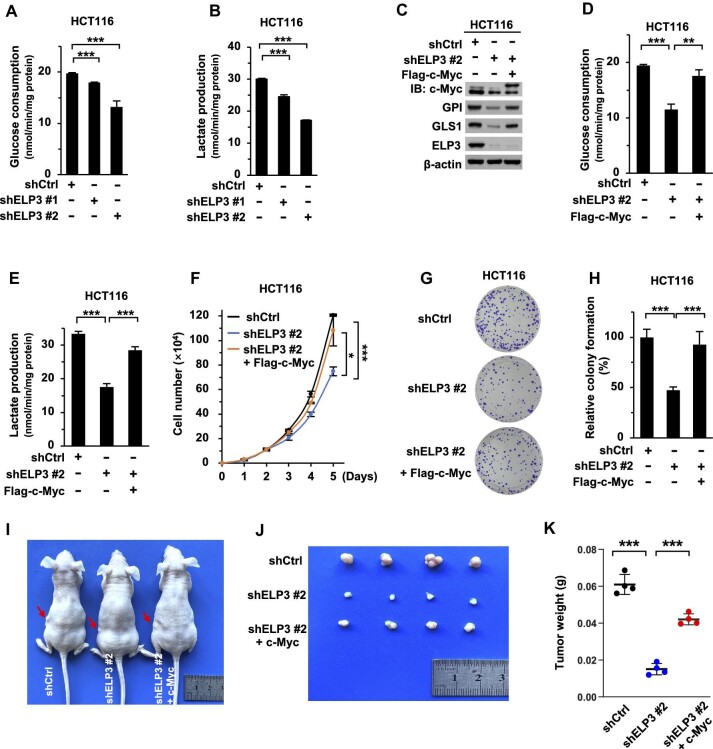
ELP3 stabilizes c-Myc to facilitate metabolic reprogramming, cell proliferation, and tumorigenesis. (**A** and **B**) Glucose consumption (**A**) and lactate production (**B**) rates in control or ELP3-knockdown HCT116 cells. (**C**–**K**) Flag-c-Myc was reconstituted in ELP3-knockdown HCT116 cells. (**C**) c-Myc, GPI, GLS1, and ELP3 protein expression. (**D**) Glucose consumption. (**E**) Lactate production. (**F**) Cell growth curves. (**G** and **H**) Colony formation assay. Cells were cultured in 6-well plates (500 cells/well) for 10 days, and the colonies were stained with crystal violet. (**I**–**K**) Xenograft tumor formation assay with 2 × 10^6^ cells per injection (*n* = 4). Data are shown as mean ± SD. Unpaired Student's *t*-test. *P < 0.05, **P < 0.01, ***P < 0.001.

Given that ELP3-mediated regulation of c-Myc and the function of c-Myc in metabolic reprogramming, we therefore explored whether ELP3 plays a role in tumor metabolism. ELP3 knockdown significantly decelerates glucose consumption and lactate production (Figure 6A–B; Supplementary Figure S2A–C). To fully address the effect on central carbon metabolism by ELP3 knockdown, we determined the levels of metabolites in glycolysis and tricarboxylic acid (TCA) cycle by liquid chromatography–mass spectrometry (LC–MS). The abundances of pyruvate, lactate, and the intermediates in TCA cycle dramatically decreased in ELP3-knockdown cells (Supplementary Figure S2D). Next, we analyzed the key metabolic enzymes in central carbon metabolism and found that glucose-6-phosphate isomerase (GPI) and glutaminase (GLS1, GAC form) display much lower levels in ELP3-knockdown cells (Supplementary Figure S2E and F). These results indicated that ELP3 facilitates the expression of GPI and GLS1 to reprogram cell metabolism.

We then determined whether ELP3-mediated metabolic reprogramming depends on c-Myc. In line with ELP3 knockdown, glucose consumption and lactate production were also significantly retarded by c-Myc knockdown (Supplementary Figure S2G and H), as well as the expression of GPI and GLS1 (Supplementary Figure S2I). We then reconstituted c-Myc expression in ELP3 knockdown cells. As expected, the rescue expression of c-Myc fully restored the protein levels of GPI and GLS1 (Figure 6C), as well as glucose consumption and lactate production (Figure 6D and E). Moreover, cell proliferation, colony formation and tumorigenic capacity were also restored when we expressed c-Myc in ELP3-knockdown cells (Figure 6F–K). In conclusion, our findings reveal that ELP3 stabilizes c-Myc to facilitate metabolic reprogramming, cell proliferation, and tumorigenesis.

### Excessive expression of ELP3 and c-Myc and its correlation in carcinoma

**Figure 7:**
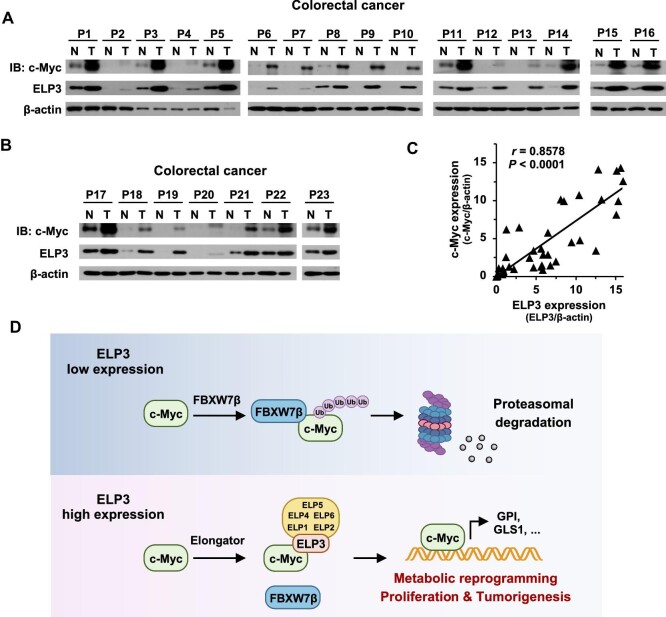
Excessive expression of ELP3 and c-Myc and its correlation with carcinoma. (**A** and **B**) Clinical tissue samples were collected from colorectal cancer patients (P, patient, *n* = 23; N, normal tissue; T, tumor tissue). c-Myc and ELP3 protein expression was determined by western blotting. (**C**) Correlation between c-Myc expression and ELP3 expression in colorectal cancer. (**D**) The model of ELP3-mediated stabilization of c-Myc in competition with FBXW7β for metabolic reprogramming, proliferation, and tumorigenesis.

To investigate the clinical relevance of the ELP3 with c-Myc, we isolated tumor tissues with corresponding normal tissues from colorectal cancer patients and analyzed the expression of ELP3 and c-Myc. Clearly, ELP3 and c-Myc were excessively expressed in colorectal tissue compared to adjacent normal tissue (Figure 7A and B). Furthermore, there is a strong correlation between c-Myc and ELP3 in colorectal cancer (Figure 7C). To widely verify the regulatory effect of ELP3 on c-Myc, we also collected the tissues of hepatocellular carcinoma and the adjacent normal tissues from 10 hepatocellular carcinoma patients. ELP3 and c-Myc were analyzed and the consistent results were observed (Supplementary Figure S3A–B). In summary, our data demonstrated that ELP3 stabilizes c-Myc by the competition with FBXW7β for c-Myc binding, thus leading to metabolic programming, cell proliferation, and tumorigenesis (Figure 7D).

## Discussion

Emerging evidence has shown that ELP3 is closely associated with malignant transformation. The importance of ELP3 in tumor progression is indicated by aberrant overexpression of ELP3 in a wide range of human tumors and the fact that ELP3 depletion abolishes tumor formation in Wnt-driven mouse model (Ladang et al., 2015; Chen et al., 2022). It is reported that ELP3 is an acetyltransferase and engaged in multiple processes of tumor progression, including resistance to apoptosis, cell proliferation, and stemness (Delaunay et al., 2016; Rapino et al., 2018). However, little is known about the carcinogenic function of ELP3 other than acetyltransferase in tumor progression. In this study, we found that ELP3 is aberrantly overexpressed in tumors and stabilizes c-Myc by direct interaction. Interestingly, ELP3-mediated stabilization of c-Myc is independent on its acetyltransferase activity, as acetyltransferase deficient mutants of ELP3 remain the intact in facilitating c-Myc expression. Mechanistically, ELP3 competes with the E3-ligase FBXW7β for binding to c-Myc, thus abolishing its proteasomal degradation. Our findings uncover the mechanism of ELP3-mediated regulation of c-Myc stability in tumor progression.

It is well accepted that tumorigenesis is a multistep process that involves the activation of pro-oncogenes and the inactivation of tumor suppressors. c-Myc is the most deregulated oncogenes described in human cancer, highlighting its importance in tumor progression. The biological characteristics that c-Myc is expressed only in cycling cells (Prochownik, 2004) and the fact that only transient inhibition is sufficient to cause tumor regression (Hart et al., 2014; Yu et al., 2016; Vaseva et al., 2018; Singh et al., 2022) make c-Myc to be an attractive target for cancer therapy. However, no inhibitor of c-Myc is yet approved for clinical use. The challenge for developing a clinical c-Myc inhibitor is at least partially owing to its intrinsically disordered nature and lacking of a binding pocket (Thomas et al., 2015; Richards et al., 2016). Nuclear location protects c-Myc protein from being targeted with monoclonal antibodies for immunotherapy which compounds the difficulty. It is worth noting that omomyc, a mini-protein derived from the b-HLH domain of c-Myc, is well developed as a c-Myc domain-negative protein to indirectly inhibit the function of c-Myc. However, the clinical efficacy of omomyc is limited by its high molecular weight and low stability (Soucek et al., 2004, 2008; Chen et al., 2018; Madden et al., 2021). In this study, we found the new mechanism of c-Myc regulation and provided evidence that ELP3 may be a potential therapeutic target for c-Myc-driven carcinomas.

## Material and methods

### Cell culture

HCT116 was obtained from the Cell Bank of the Chinese Academy of Sciences (Shanghai). HeLa and HEK-293T were purchased from the American Type Culture Collection (ATCC). All cell lines were stored in our laboratory cell bank and prepared for use. Each of cell line was cultured in Dulbecco's modified Eagle's medium (DMEM; Gibco, #11965) supplemented with 10% fetal bovine serum (FBS) and maintained in a humidified incubator containing 5% CO_2_ at 37°C.

### Plasmids construction

Full-length cDNAs encoding ELP1–ELP6, c-Myc, and p300 were purified and inserted into pBOBI and pcDNA3.3 vector. ELP3 Y529A was established by a mutated-site primer-mediated polymerase chain reaction (PCR) mutagenesis method using DNA polymerase (PrimeSTAR, TaKaRa). All ELP3 and c-Myc deletion forms were constructed using PCR based on ligase-independent cloning methodology. The pLKO.1-puro vector was used to construct shRNA plasmids. The nucleotide target sequences for shRNAs are as follows: shCtrl: 5′-CGACCACATGAAGCAGCACGA-3′; shELP3 #1: 5′-CCTACAGACAAGACACCGAAT-3′; shELP3 #2: 5′-CGCAAGGTCTTGATGCCCAAGTTAA-3′; shELP4: 5′-GAGAGAAACTAACCCATTGTA-3′; shELP5: 5′-CCACATCTTCTATGAGCCAGA-3′; shFBXW7β: 5′-ATGGGTTTCTACGGCACATTA-3′; shMYC: 5′-GATGAGGAAGAAATCGATG-3′.

### Transfection, lentivirus package, and infection

Polyethylenimine (PEI; MW: 40000; Sigma) was used for transient transfection in HEK-293T cells. Lentivirus was also packaged in HEK-293T cells. Briefly, equal plasmids and lentivirus elements (PMDL:VSVG:REV = 5:3:2) were transfected using lipofectamine transfection reagent (Thermo Scientific, #R0532) according to the manufacturer's instructions. After packing 48 h, the virus supernatant was collected by centrifugation (3000× *g*, 5 min) and stored at −80°C prepared for infection. For lentivirus infection, cell confluency should be controlled at 30%–50%. Before infection, the cultured medium was replaced with fresh DMEM supplemented with polybrene (10 μg/ml). Cells were infected with virus for the next 24 h. All experiments were performed after expression or knockdown efficiency were examined by western blotting.

### Antibodies and reagents

Rabbit anti-c-Myc (10828-1-AP), rabbit anti-TPI (10713-1-AP, 1:1000 for IB), rabbit anti-PGAM1 (16126-1-AP, 1:1000 for IB), rabbit anti-PGAM2 (15550-1-AP, 1:1000 for IB), and rabbit anti-FBXW7 (28424-1-AP, 1:2000) were purchased from Proteintech. Mouse anti-HA (sc-7392, 1:1000 for IB), rabbit anti-GPI (sc-33777), rabbit anti-GAPDH (sc-25778, 1:1000 for IB), rabbit anti-enolase (sc-15343, 1:1000 for IB), goat anti-PGK1 (sc-17943, 1:500 for IB), and mouse anti-p53 (sc-126; 1:1000 for IB) were purchased from Santa Cruz Biotechnology. Mouse anti-FLAG (M2) (M8823, 1:5000) and mouse anti-β-actin (A5441, 1:2000 for IB) were purchased from Sigma. Rabbit anti-HK1 is homemade and preserved in our lab. Rabbit anti-ELP3 (A0020) and rabbit anti-ELP5 (A14862, 1:1000 for IB) were obtained from ABclonal Technology. Rabbit anti-ELP4 (ab133687, 1:1000 for IB) was purchased from Abcam. Rabbit anti-p-T58-c-Myc (#46650, 1:1000 for IB) and rabbit anti-p-S62-c-Myc (#13748, 1:1000 for IB) were purchased from Cell Signaling Technology. ELP3 Δ1–81 was detected by primary ELP3 antibody (#22597, 1:1000 for IB) from SAB signalway company.

### Glucose consumption and lactate production

For glucose consumption and lactate production measurement, cells at ∼80% confluency were rinsed with warmed phosphate buffered saline (PBS) and then cultured in fresh DMEM containing 10% FBS and 5 mM glucose for another 4 h. The cell-cultured medium was collected, and glucose and lactate contents were determined by glucose kit (Rongsheng Biotech) and lactate kit (Co-Health Bio-technologies Inc.), respectively. Fresh medium was used as a control for the computation of glucose consumption and lactate production (normalized to protein).

### IP and western blotting

Cells were scraped in lysis buffer (20 mM Tris–HCl, pH 7.4, 150 mM NaCl, 1 mM EDTA, 1 mM EGTA, 1% Triton, 2.5 mM sodium pyrophosphate, 1 mM β-glycerolphosphate, 1 mM sodium orthovanadate, 1 μg/ml leupeptin, and 1 mM phenylmethylsulfonyl fluoride), followed by sonication and centrifugation at 20000× *g* for 12 min at 4°C. For IP, 10% supernatant was collected as input (total lysate). Residual lysate was subjected to agarose beads and rotated for another 3 h. Generally, anti-Flag M2 beads (Sigma-Aldrich) were used for Flag-tagged proteins and A/G beads (Thermo Scientific) with the indicated antibody for the specific protein. The immunoprecipitates were collected and then washed 3 times with fresh lysis buffer by centrifugation at 3000× *g* for 1 min. Total input and immunoprecipitates were added with sodium dodecyl sulphate (SDS) loading buffer, boiled for 10 min, and subjected to SDS–polyacrylamide gel electrophoresis (PAGE) followed by transfer to polyvinylidene fluoride (PVDF) membranes (Roche). PVDF membranes were incubated with specific antibodies according to the manufacturer's instructions after blockade with 5% milk (*w*/*v*). Proteins were visualized by chemiluminescence system with films. Relative protein expression levels (normalized to control) were determined according to the optical density integrity (ODI) with the ImageJ software (NIH).

### Protein purification and GST-pulldown assay

Plasmids encoding GST, GST-ELP3 1–81, and MBP-His-c-Myc 1–143 were transformed into BL21 *Escherichia coli* competent cells. The *E. coli* cells were grown in shaking lysogeny broth (LB) medium to OD_600_ value between 0.7 and 0.8. Next, isopropyl-β-D-thiogalactoside (IPTG, 500 μM) was added to stimulate protein expression for 18 h. Proteins were purified with glutathione sepharose beads or Ni-NTA beads. GST-pulldown assay was performed by incubating MBP-His-c-Myc 1–143 with GST-ELP3 1–81 (GST as control) in the buffer (50 mM Tris–HCl, pH 7.6, 120 mM NaCl, 5 mM MgCl_2_, 0.5% nonidet P-40 (NP-40), and 1 mM EDTA) for 3 h at 4°C, followed by incubation with glutathione sepharose beads. The precipitates were washed extensively with the above buffer and analyzed by SDS–PAGE and Coomassie brilliant blue staining with a Bio-Rad imaging system.

### Cell growth curves and xenograft tumor

Cell lines were constructed by infection with lentivirus. Then, cells were trypsinized and 1 × 10^4^ cells were seeded into 6-well plates individually (*n* = 3). Cell numbers were counted at each 24 h and cell growth curves were depicted according to the results. For the tumor xenograft assay, lentivirus was packaged and infected to establish stable cell lines (as described in the section of lentivirus package and infection). After the check of knockdown or overexpression of specific protein by western blotting, 1 × 10^6^ cells were resuspended in 200 μl PBS and subcutaneously injected to the right or left flanks of 8-weeks old nude mice. Mice were sacrificed after one month of injection and the tumors were isolated and weighted as shown in results.

### CCK-8 assay for cell viability

Cells were counted and seeded into 96-well plates (*n* = 3) individually, followed by CCK-8 assays in each 24 h according to manufacturer's instructions (YEASEN, 40203ES76). A volume of 10 μl of the CCK-8 solution was added to each well for 30 min of incubation and the absorbance (OD_450_) was read by a microplate reader (Tecan Spark). Cell viability was analyzed on the basis of OD_450_ absorbance.

### Colony formation assay

Cell number (as described in figure legend) was calculated and seeded in 6-well plates (*n* = 3), followed by cell culture for several days. After crystal violet staining, the colony number was calculated for analysis.

### Quantitative real-time PCR (qRT-PCR)

Total RNA from HCT116 and HeLa cells was extracted using the TRIzol™ Reagent (Invitrogen) and quantified by Nano Drop spectrophotometer (ND-1000, Thermo Scientific). Reverse transcription was performed for cDNA synthesis using PrimeScript™ RT Reagent Kit with gDNA Eraser (TaKaRa). qRT-PCR was carried out to determine the mRNA level by GoTaq® qPCR kit (Promega) on an ABI Prism-7500 sequence detection system (ABI, Applied Biosystems). The relative expression levels of mRNAs were normalized to β-actin. Primers used in qRT-PCR were listed as follows: ELP3 forward primer: 5′-AACTGATTGAAGCCCACGA-3′; ELP3 reverse primer:5′-GCAGCAATGATATCCACCAG-3′; c-Myc forward primer: 5′-TTCTGTGGAAAAGAGGCAGG-3′; c-Myc reverse primer: 5′-TGCGTAGTTGTGCTGATGTG-3′; β-actin forward primer: 5′-TCCATCATGAAGTGTGACGT-3′; β-actin reverse primer: 5′-TACTCCTGCTTGCTGATCCAC-3′.

### Immunofluorescence

Cells were cultured on slices in 6-well plates and fixed with 4% paraformaldehyde for 10 min at room temperature. Then, the cells on slice were rinsed with PBS and permeabilized with 0.2% Triton X-100 PBS solution for 5 min, followed by blockade with 5% BSA for 1 h. Cells were rinsed once with PBS and were incubated with primary antibodies according to the manufacturer's instruction. Then, cells were rinsed with PBS three times, and incubated with secondary antibody (Alexa-Fluor 488-conjugated and 555-conjugated antibodies) for 1 h. For nuclear staining, 4′,6-diamidino-2-phenylindole (DAPI) was incubated at 37°C for 10 min. Zeiss 780 confocal microscope was used to obtain immunofluorescence images processed with Adobe Photoshop for publishment.

### Cytosol and nuclear extraction

Cells were seeded at 80% confluency 24 h before extraction (35 mm dish). For details, cells were added with 120 μl Buffer A (10 mM Hepes, pH 7.9, 10 mM KCl, 0.1 mM EDTA, 0.1 mM EGTA, 1 mM DTT, 0.15% NP-40, and 1% cocktail) and kept on ice for 10 min (20 μl cell lysate was collected as input). The lysate was centrifuged at 12000×*g* for 30 sec. The supernatant fraction was collected as cytosolic extraction. The nuclear pellet was washed 3 times with Buffer A and dispersed in Buffer B (2 mM Hepes, pH 7.9, 400 mM NaCl, 1 mM EDTA, 1 mM EGTA, 1 mM DTT, 0.5% NP-40, and 1% cocktail). After sonication (10 sec, 3 times), the nuclear lysate was centrifuged at 20000× *g* for 15 min. The supernatant was collected as nuclear fraction. The expression of ELP3, c-Myc and FBXW7β in cytosol and nuclear extraction were analyzed by western blotting.

### LC–MS

The sample preparation of LC–MS for metabolic analysis was performed as previously described (Chaneton et al., 2012). To analyze the metabolites, cells were seeded at 60%–70% confluency and the medium was changed with complete medium for another 24 h. The cells were washed with pre-cold PBS and quenched by ice-cold 80% methanol solution. The mixture was vortexed for 20 sec to totally release the cell components and centrifuged at 12000× *g* for 30 min at 4°C. The supernatant was collected and dried by a vacuum centrifugal concentrator at 4°C. The dried cell extracts were dissolved in 50% acetonitrile solution, prepared for subsequent LC–MS analysis.

For metabolites analysis, LC with SCIEX ExionLC AD was prepared and all chromatographic separations were performed with a Millipore ZIC-pHILIC column (5 μm, 2.1 × 100 mm internal dimensions, PN: 1.50462.0001). The column was maintained at 40°C and the injection volume of all samples was 2 μl. The mobile phase consisted of 15 mM ammonium acetate and 3 ml/L ammonium hydroxide (>28%) in LC–MS grade water (mobile phase A) and LC–MS grade 90% (*v*/*v*) acetonitrile-HPLC water (mobile phase B) run at a flow rate of 0.2 ml/min. Analysts were separated with the following gradient program: 95% B held for 2 min, increased to 45% B in 13 min, held for 3 min, and the post-time was set to 4 min.

The gradient was 95% B for 2 min, 45% B within 13 min (linear gradient), maintained for 3 min, directly 95% B and maintained for 4 min. The flow rate was 0.2 ml/min. The quadrupole linear trap (QTRAP) mass spectrometer using a Turbo V ion source. The ion source was run in negative mode with a spray voltage of 4500 V, Gas1 40 psi and Gas2 50 psi and Curtain gas 35 psi, Metabolites were measured using the multiple reactions monitoring (MRM) mode, The relative amounts of metabolites were analyzed by MultiQuant Software (AB SCIEX).

### Statistical analysis

Data are presented as mean ± SD of at least three independent experiments. Student's *t*-test (unpaired, two-tailed) was used to determine the statistical difference between two groups. n.s., no significance (*P* ≥ 0.05); *P < 0.05, **P < 0.01, ***P < 0.001.

## Supplementary Material

mjad059_Supplemental_File

## Notes

**Author contributions:** W.Z., C.O., and C.H. contributed equally to this work. W.Z. and C.O. designed the research; W.Z., C.O., C.H., J.Z., and Q.X. took responsibility for the experiments; W.Z. drafted the manuscript; W.Z. and C.O. performed data analysis; F.Z., H.W., F.L., and J.W. provided scientific and technical support; Z.W., B.J., and Q.L. critically revised the manuscript. All authors contributed to writing the manuscript and have given approval for the final version of the manuscript.
