# Supplementary material for: ELP3 stabilizes c-Myc to promote tumorigenesis
Source: J Mol Cell Biol. 2023 Sep 28;15(9):mjad059. doi: 10.1093/jmcb/mjad059 (PMC11054291; doi:10.1093/jmcb/mjad059)
Supplement: mjad059_Supplemental_File [file mjad059_supplemental_file.pdf]

Supplementary Figures

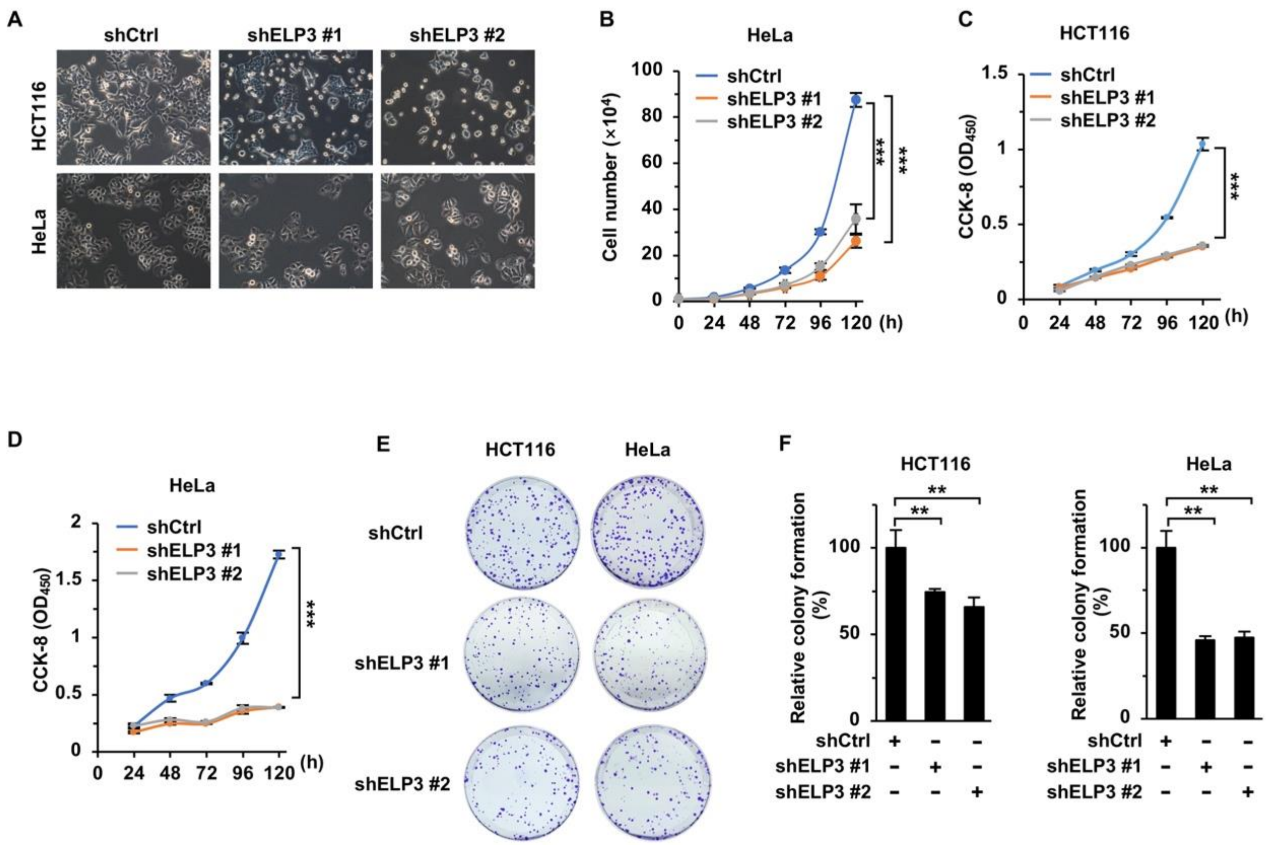

Supplementary Figure S1. ELP3 is associated with cell proliferation. (A) Images of HCT116 and HeLa were captured by Olympus microscope after 3 days of virus infection. **(B)** 1×10^4 cells (pLKO.1 scramble shRNA as control, shCtrl) were seeded in 6-well plates. The cell growth curves were depicted based on the cell-counting results as indicated time (n=3). **(C** and **D)** 2,000 HCT116 (C) and HeLa (D) cells with shCtrl or shELP3 were seeded in 96-well plates. CCK-8 reagent was added after cell culture as indicated times. The absorbance of OD_{450} was read after 30 min (n=3). **(E** and **F)** ELP3 in HCT116 and HeLa was knocked down and 500 cells were seeded in 6-well plates and colony number was calculated after 10 days using crystal violet staining.

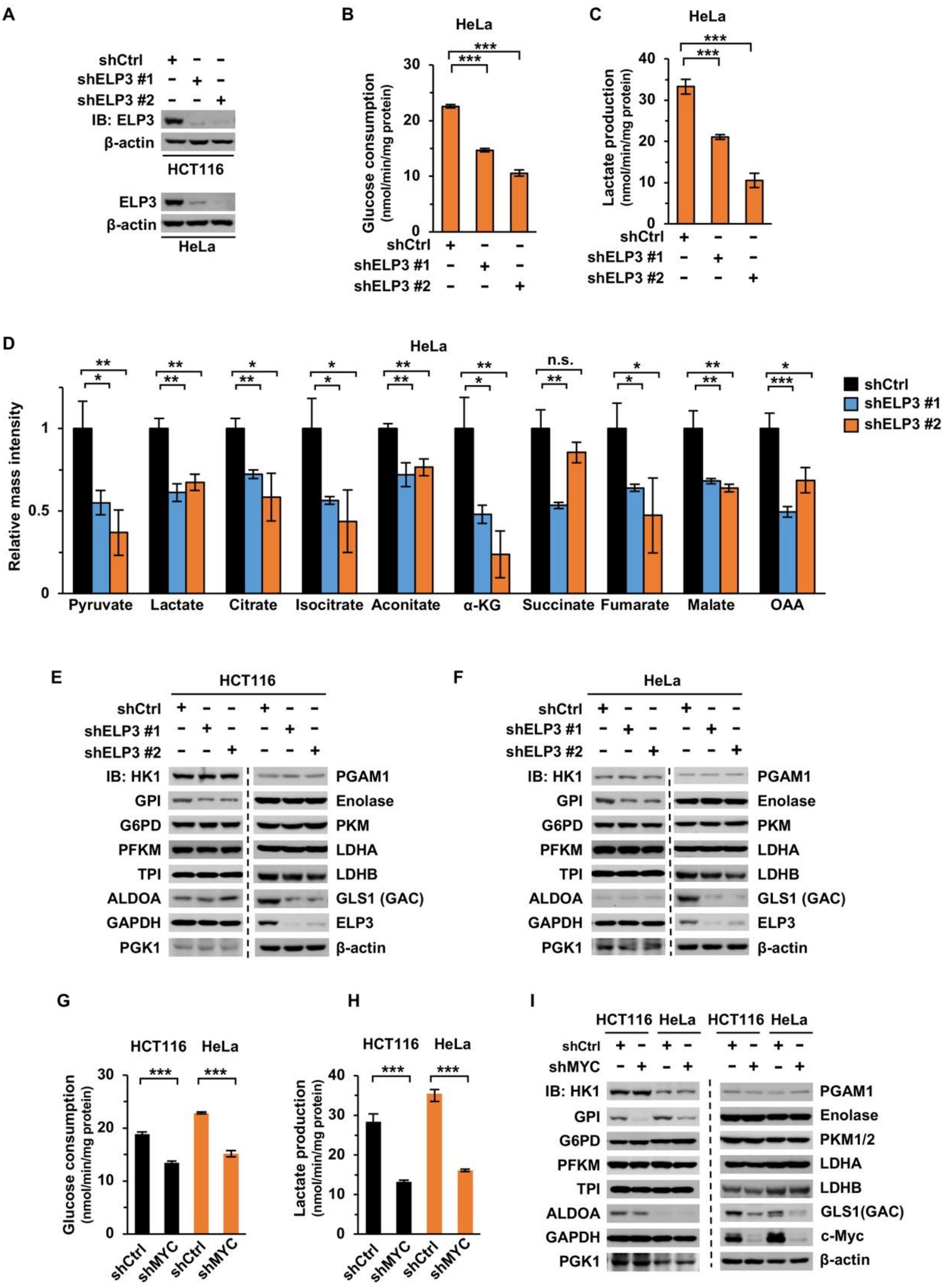

Supplementary Figure S2. ELP3 and c-Myc facilitate glycolysis and glutaminolysis. (A) HCT116 and HeLa were infected with lentivirus expressing shCtrl or shELP3 (shELP3 #1 and shELP3 #2). After infection 3 days, cells were harvested and subjected to SDS-PAGE followed by analysis of ELP3-

knockdown efficiency. (**B** and **C)** ELP3-knockdown HeLa cells were seeded in 6-well plates to culture up to 80% confluency. Cells were rinsed with PBS and cultured in DMEM containing to 5 mM glucose for another 4 h. Glucose and lactate in cell-cultured media were determined by glucose kit or lactate kit individually. And glucose consumption rate (B) and lactate production rate (C) were calculated and normalized to protein mass. **(D–F)** HeLa shCtrl and shELP3 cells were incubated with complete medium and the metabolites in glycolysis and TCA cycle were determined by LC-MS (D). Enzymes in glycolytic flux and glutamine metabolism were detected by WB when ELP3 was knocked down in HCT116 (E) and HeLa (F). **(G–I)** Glucose consumption (G) and lactate production (H) were analyzed in c-Myc-knockdown cells via glucose kit and lactate kit respectively. Enzymes in glycolytic flux and glutamine metabolism were detected by WB (I).

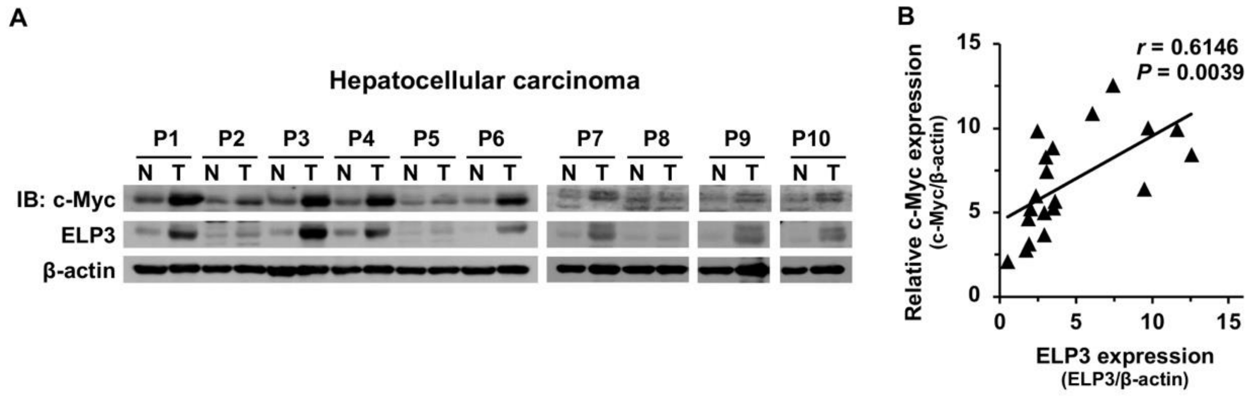

Supplementary Figure S3. Excessive expression of ELP3 and c-Myc and its correlation in hepatocellular carcinoma. (A and **B)** Tissues from hepatocellular carcinoma (HCC) patients were collected (P: patient, n=10), followed by determination of c-Myc and ELP3 expression (A) and analysis of the correlation of c-Myc with ELP3 (B).
